# Posterior reversible encephalopathy syndrome due to nonadherence to antihypertensive treatment: A case report from Nepal

**DOI:** 10.1002/ccr3.8393

**Published:** 2024-01-02

**Authors:** K.C. Priyanka, Ayush Anand, Salman Haidar Husain, Urza Bhattarai, Sanjib Kumar Sharma

**Affiliations:** ^1^ Department of Internal Medicine B. P. Koirala Institute of Health Sciences Dharan Nepal; ^2^ B. P. Koirala Institute of Health Sciences Dharan Nepal

**Keywords:** case report, Nepal, nonadherence, posterior reversible encephalopathy syndrome

## Abstract

**Key Clinical Message:**

Posterior reversible encephalopathy syndrome may occur secondary to abrupt cessation of antihypertensive therapy. A gradual reduction in blood pressure and counseling regarding medication adherence are crucial to prevent adverse consequences.

**Abstract:**

Posterior reversible encephalopathy syndrome (PRES) is a reversible clinical radiographic syndrome with headache, hypertensive encephalopathy, seizures, and visual disturbances as common modes of presentation. PRES can be attributed to several risk factors. We reported the case of a 66‐year‐old Asian female with PRES following nonadherence to antihypertensive treatment. Initially, her computed tomography scan of the head was normal. After 48 h, we again ordered a head CT scan, which showed lesions suggestive of hypertensive encephalopathy. We immediately reduced 20%–25% of mean arterial pressure, followed by a gradual blood pressure lowering to avoid adverse consequences. We did a follow‐up CT scan of the head at 2 weeks, showing the resolution of early lesions. Hence, we made a diagnosis of PRES. In these patients, it is crucial to ensure medication adherence to avoid complications.

## Graphical abstract

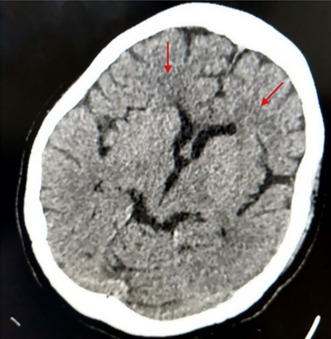

## 1 INTRODUCTION

Posterior reversible encephalopathy syndrome (PRES) was first described by Hinchey et al.^1^ It is a reversible clinical radiographic syndrome with varying severity.^2^, ^3^, ^4^ The patients can present with headaches, hypertensive encephalopathy, seizures, and visual disturbances.^1^, ^2^, ^3^ In these patients, it is crucial to take a detailed history, and investigations are necessary to rule out associated medical conditions (Table 1). For diagnosing PRES, the preferred modality is magnetic resonance imaging (MRI) of the brain.^3^, ^5^, ^6^ In limited resource settings, a computed tomography (CT) scan of the head and clinical evaluation are sufficient to reach a diagnosis.^3^ PRES is commonly associated with hypertensive encephalopathy.^3^, ^4^ An acute rise in blood pressure leading to PRES can be attributed to various medical conditions, such as eclampsia and kidney injury. Various factors can be attributed to the development of this syndrome (Table 1). In patients with PRES, cautious lowering of blood pressure is advised with vigilant monitoring to prevent adverse events.^7^ Abrupt cessation of antihypertensive therapy usually leads to sympathetic overactivity in 2–3 days, with no immediate consequences.^8^ Also, it does not lead to the return of hypertension in one‐fourth of the patients.^9^ However, sometimes adverse effects do occur.^9^ Herein, we report the case of PRES following the abrupt cessation of antihypertensive medication in a 66‐year‐old female from Nepal.

**TABLE 1:** Conditions associated with posterior reversible encephalopathy syndrome.^1^, ^2^, ^3^, ^4^, ^10^, ^11^, ^12^, ^13^

Eclampsia and pre‐eclampsia
Acute and chronic kidney disease
Infections/Sepsis/Shock
Chemotherapeutic agents
Autoimmune diseases
Blood transfusion associated Transplantation associated
Hemolytic uremic syndrome
Vasculitis
Hypertensive encephalopathy

## 2 CASE PRESENTATION

### 2.1 History

A 66‐year‐old female presented to the emergency department (ED) with altered sensorium, weakness in upper bilateral limbs, and slurring speech for 1 h. There was no history of seizure, tongue bite, or incontinence. She has been under medication for type 2 diabetes mellitus (Tab Metformin 1 g PO OD), primary hypertension (Tab Amlodipine 5 mg PO OD and Tab Losartan 50 mg PO OD), and primary hypothyroidism (Tab Levothyroxine 25 mcg PO OD) for the past 10 years. On further inquiry, she revealed that she stopped taking antihypertensives 1 week ago. She did not smoke or consume alcohol. Her family history and drug history were unremarkable. Her baseline blood pressure was 140/80 under dual antihypertensive therapy (amlodipine 5 mg and losartan 50 mg).

### 2.2 Physical examination

On presentation, her blood pressure was 210/110 mmHg, pulse was 115 beats per minute, respiratory rate 18 cycles/min, SpO2 was 95% in room air, and Glasgow Coma Scale (GCS) score was 13/15.^14^ Her random blood glucose level was 140 mg/dL. Immediate electrocardiogram was done, which was normal. Rapid diagnostic kit for cardiac troponin was negative. On central nervous system (CNS) examination, higher motor function was intact. The motor power of the left and right upper limbs were 3/5 and 5/5, respectively. Also, the motor power of the left and right lower limbs were 5/5 and 4/5, respectively. No visual deficits were reported. The rest of her CNS and systemic examinations were unremarkable. Her ophthalmological examination showed grade 1 hypertensive retinopathy. In our patient, we considered differentials such as stroke, transient ischemic attack, encephalitis, and cerebral venous thrombosis.

### 2.3 Investigations

Her initial laboratory investigations revealed anemia (Hb = 9.6 g/dL), hypertriglyceridemia (triglyceride = 430.67 mg /dL), hypothyroidism (TSH = 9.7 IU/mL), and poor glycemic control (HbA1c = 9.31%). Her echocardiography report showed grade 1 left ventricular diastolic dysfunction. Since the clinical evaluation was suggestive of neurological involvement, we did an emergency CT scan of the head, which was normal. Also, we did a CT pulmonary angiogram, which was normal. Since her head CT scan was normal, ischemic stroke and cerebral venous thrombosis were less likely. However, ischemic stroke was still a possibility due to early CT imaging.

### 2.4 Management

Initially, we managed the patient as a case of hypertensive encephalopathy. The patient was started on labetalol 20 mg IV stat, which did not lead to a significant decrease in mean arterial pressure (MAP). So, we gave intravenous (IV) injection of nitroglycerin to target an immediate 20% decrease in MAP. Her GCS dropped to 8/15 over 30 min, for which she was shifted to the intensive care unit (ICU) and intubated.

**FIGURE 1:**
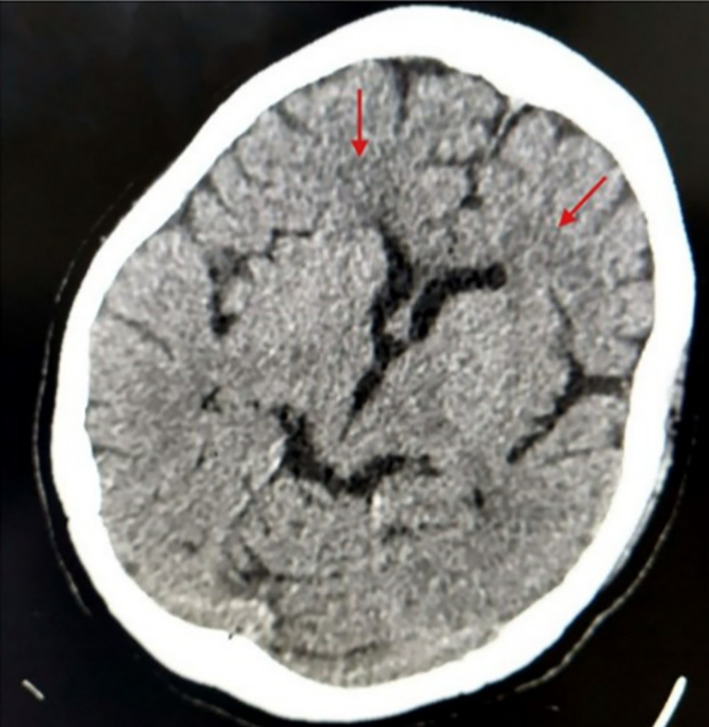
Computed tomography scan of the head showing white matter hypodensities (red arrow) in bilateral frontal lobes.

After shifting to ICU, her blood pressure was 160/90 mm Hg. Following this, we managed hypertension, hypothyroidism, and T2DM. During the second day of her stay in the ICU, we did a CT scan of the head, which showed bilateral symmetrical white matter hypodensities in bilateral frontal lobes (Figure 1). Initially, we could not do a lumbar puncture as the patient's condition deteriorated suddenly. During her ICU stay, we did a lumbar puncture, which was normal, and ruled out encephalitis. Also, her electroencephalogram (EEG) was not suggestive of seizure disorder. Based on the detailed evaluation, we diagnosed the patient with hypertensive encephalopathy. We used tablet amlodipine 5 mg twice daily per nasogastric tube (NG), tablet telmisartan 40 mg once daily per NG to treat hypertension, and thyroxine 62.5mcg once daily per NG for hypothyroidism. Also, we gave insulin glargine for diabetes mellitus, which was optimized based on her blood sugar levels. Along with this, intravenous fluids were given as supportive treatment for her daily fluid requirement. Her GCS raised to 15/15 on day 10 in ICU, and she was extubated. After 18 days of ICU stay, she was shifted to the medicine ward. She stayed in the medicine ward for 7 days. Her blood pressure at discharge was 140/80 mm of Hg. The power of her right upper limb was 3/5, and that of her left limb was 3/5. The patient was educated about the adverse effects following the abrupt stoppage of antihypertensive medication and was counseled for medication adherence. We prescribed tablet amlodipine 5 mg per oral twice daily and telmisartan 40 mg per oral once daily for hypertension. Also, we encouraged early ambulation and advised limb physiotherapy. On her follow‐up after 2 weeks, a CT scan of her head was done, which showed the resolution of previous hypodense lesions. On her monthly follow‐up, the patient adhered to medications, and her blood pressure was 140/80 mm of Hg, with no complications.

## 3 DISCUSSION

**FIGURE 2:**
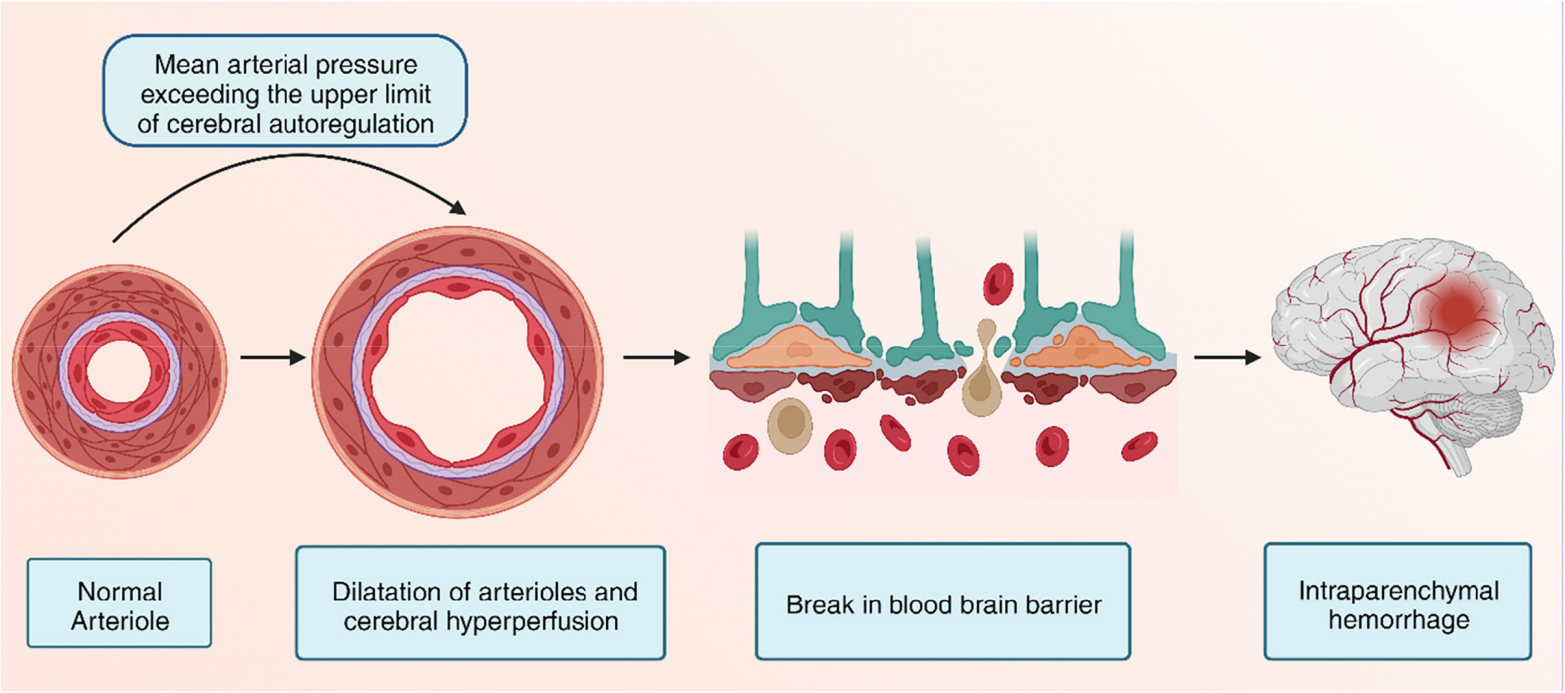
Failure to autoregulate cerebral blood flow leads to intracranial hemorrhage [created with Biorender.com].

**FIGURE 3:**
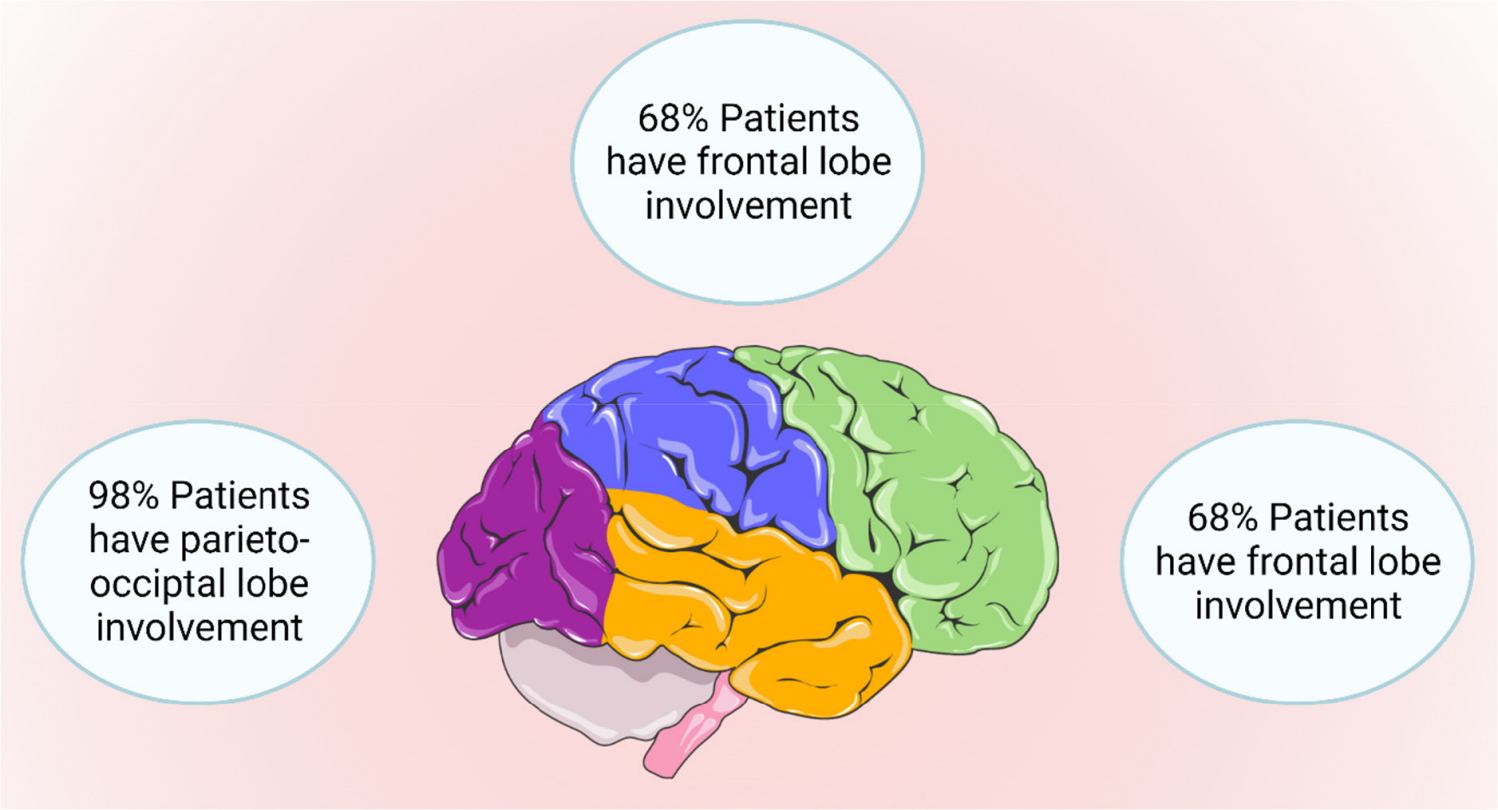
Commonly involved areas of the brain in posterior reversible encephalopathy syndrome [created with Biorender.com].

The exact incidence of PRES syndrome is unknown. The name is a misnomer as the condition may sometimes not be reversible and may not only involve the posterior cerebrum.^1^, ^3^ Though the exact pathophysiology is unknown, three hypotheses have been proposed. The most widely accepted hypothesis (Figure 2) is that the failure of autoregulation of cerebral blood flow leads to infarcts in the brain and marked hypertension, particularly in patients with chronic hypertension.^3^, ^13^ In these cases, it is essential to control hypertension rationally, as a sudden drop in blood pressure can lead to cerebral ischemia.^3^ Other hypotheses suggest that cerebral blood flow dysregulation can lead to focal vasoconstriction and vasogenic edema. Alternatively, endothelial dysfunction may lead to PRES in settings with pre‐eclampsia or cytotoxic medications.^1^, ^3^, ^13^ These most commonly reported clinical signs and symptoms include encephalopathy, seizure disorder, headache, and visual disturbances.^3^ Imaging modalities (Figure 3) reveal lesions mainly in the parieto‐occipital and frontal lobes.^1^, ^3^, ^5^, ^6^, ^13^ Our patient presented with hypertensive encephalopathy after 1 week of cessation of antihypertensive medication. Though the preferred modality is an MRI of the brain, a head CT scan is sufficient for diagnosing hypertensive encephalopathy in low‐resource settings.^3^ The initial CT scan was normal. However, a CT scan after 48 h revealed involvement in the frontal lobe. Based on clinical presentation and radiological evaluation, we made a provisional diagnosis of hypertensive encephalopathy. Based on the International Society of Hypertension guidelines, initial management was focused on the immediate reduction of 20%–25% of mean arterial pressure using labetalol.^7^ Since the patient did not respond to labetalol, we switched to nitroglycerin infusion for adequate blood pressure control. As soon as the blood pressure was controlled, we switched to antihypertensives via NG as the patient was intubated. Gradually, the blood pressure was controlled. On follow‐up after 2 weeks, a head CT scan was normal. This pointed towards the diagnosis of PRES.

A meta‐analysis by Lee et al.^15^ involving 27 million hypertensive patients revealed that approximately 27%–40% were nonadherent to medications, with higher prevalence in low‐ to middle‐income countries. The patients who were nonadherent to drugs were twice as likely to develop complications.^15^ Additionally, these patients had a 38% increased risk of hospitalization and mortality.^15^ Our case suggested the development of PRES following nonadherence to antihypertensive treatment. In these cases, it is crucial to counsel the patients regarding medication adherence to prevent adverse consequences.

## 4 CONCLUSION

Our case highlighted PRES as a consequence to abrupt cessation of antihypertensive therapy. Evidence‐based management with a gradual reduction in blood pressure is crucial to prevent adverse consequences. Additionally, it is critical to counsel the patients regarding medication adherence to avoid complications in the future.

## AUTHOR CONTRIBUTIONS

**Priyanka K.C.:** Conceptualization; data curation; project administration; supervision; writing – original draft; writing – review and editing. **Ayush Anand:** Conceptualization; data curation; project administration; supervision; writing – original draft; writing – review and editing. **Salman Haidar Husain:** Data curation; writing – original draft; writing – review and editing. **Urza Bhattarai:** Data curation; project administration; supervision; writing – review and editing. **Sanjib Kumar Sharma:** Data curation; project administration; supervision; writing – review and editing.

## FUNDING INFORMATION

The author(s) did not receive any funding for this work.

## CONFLICT OF INTEREST STATEMENT

The author(s) have no conflict of interest to declare.

## ETHICS STATEMENT

Our institution does not require ethical approval for reporting individual cases or case series.

## CONSENT

Written informed consent was obtained from the patient(s) for their anonymized information to be published in this article. The patient had the decisional capacity to provide written informed consent.

## Data Availability

All relevant data pertaining to this case report is available within the manuscript.
